# Predictors of pediatric tuberculosis in public health facilities of Bale zone, Oromia region, Ethiopia: a case control study

**DOI:** 10.1186/s12879-018-3163-0

**Published:** 2018-06-04

**Authors:** Bereket Gebremichael, Tsega-Ab Abebaw, Tsedey Moges, Admas Abera Abaerei, Nadia Worede

**Affiliations:** 1 College of Medicine and Health Sciences, Allied School of Health Science, Addis Ababa University, Addis Ababa, Ethiopia; 2 Department of Occupational Health, Higher Education Relevance and Quality Agency, Addis Ababa, Ethiopia; 3 Ethiopian Public Health Institute, Food Science and Nutrition Directorate, Addis Ababa, Ethiopia; 4 College of Health and Medical Sciences, School of public health, Haramaya University, Harar, Ethiopia; 5 Addis Ababa, Ethiopia; 6 Harar, Ethiopia

**Keywords:** Tuberculosis, Pediatrics, Predictors of pediatric TB, Bale zone

## Abstract

**Background:**

Tuberculosis is among the top ten cause of death (9th) from a single infectious agent worldwide. It even ranks above HIV/AIDS. It is among the top 10 causes of death among children. Globally there are estimates of one million cases of TB in children, 76% occur in 22 high-burden countries among which Ethiopia ranked 8th. Despite this fact, children with TB are given low priority in most national health programs. Moreover reports on childhood TB and its predictors are very limited. Therefore this study aimed to assess predictors of pediatric Tuberculosis in Public Health Facilities.

**Methods:**

Unmatched case control study among a total samples of 432 (144 cases and 288 controls) were done from August to December 2016 in Bale zone, South East Ethiopia. Pediatric TB patients who attended health facilities for DOTS and those who attended health facilities providing DOTS service for any health problem except for TB were the study population for cases and controls, respectively. For each case two consecutive controls were sampled systematically. Data were collected using pretested and structured questionnaire through face to face interview with parents. Binary and multivariable logistic regression analyses were employed to identify predictors of Tuberculosis.

**Result:**

Among cases there were equal number of male and female 71(50%). However among control 136 (47.9%) were male and the rest were female. The mean (standard deviation) of age among cases was 8.4 (±4.3) and controls were 7.3 (±4.1). The odds of TB were 2 times (AOR, 95% CI = 1.94(1.02–3.77)) more likely among 11–15 age group children when compared with children of age group ≤5. HIV status of the child, children who were fed raw milk and absence of BCG vaccination were the other predictors of pediatric TB with AOR 13.6(3.45–53.69), 4.23(2.26–7.88), and 5.46(1.82–16.32) respectively.

**Conclusion:**

Children who were not BCG Vaccinated were at risk of developing TB. Furthermore, HIV status, age of the child and family practice of feeding children raw milk are the independent predicators of pediatric TB in the study area.

## Background

Tuberculosis (TB) is a chronic infectious disease caused by *Mycobacterium tuberculosis* (*MTB*). It typically affects the lungs (pulmonary TB) but can affect other parts of the body as well (extra pulmonary TB) [1]. If properly treated it is medicable in virtually all cases but it remains as one of the major public health problems worldwide. It is a major cause of disease and death worldwide and has infected around one-third of the world population [2]. In 2015, there were an estimated 10.4 million new (incident) TB cases worldwide among which 1.0 million (10%) were children. People living with HIV accounted for 1.2 million (11%) of all new TB cases. WHO South-East Asia and African region contributed for higher number (greater than 70%) of incident cases [3, 4].

Even though the proportion of TB in children is smaller, the urgency of the problem of TB in children cannot be underestimated. They are at high risk of developing TB due to their developing immune system. The majority of them tends to develop severe and disseminated form of TB within a year or two and could die even before their diagnosis [5]. Every day around 200 children die due to tuberculosis: disease that can be prevented and treated [6]. According to the 2015 WHO estimate and study on the global burden of tuberculosis mortality in children, the annual burden of childhood mortality were over 200,000 worldwide [7, 8]. Given the challenge in diagnosing childhood TB, the actual burden of TB in children is likely higher than the estimate [6, 9].

In the last 10 years tuberculosis has reemerged as a major worldwide public health hazard with increasing incidence among adults and children. Even though cases among children are not as prevalent as adult, infected children are a reservoir from which many adult cases will arise [4]. Ethiopia is among the 22 high burden countries ranked eighth in the world with an estimated TB incidence (all forms) of 378 new cases per 100,000 persons. It is not surprising if the country is also among high burden countries with childhood TB. Children are at high risk of developing TB due to their un matured immune system. In Ethiopia, close to 13% of notified TB cases were children [10].

TB is one of the top ten diseases that cause childhood morbidity and mortality in Oromia regional state and specifically in Bale Zone. In 2014/15 G.C around 3345 population were on DOTS among which pediatric cases were around 628 which account around 18.77% [11].

According to studies done in different areas, different factors that are directly and indirectly related to poverty like malnutrition, living standard and style, some demographic characteristics are assumed to be independent predictors of childhood tuberculosis [12, 13–16]. Even though there are studies that focus on childhood TB done in Ethiopia, they only focus on prevalence and treatment outcome and most of the studies were cross sectional in their design [10, 17, 18, 19]. Very few studies were done on risk factors of TB but they were done on the general population or focus on the adult population even though the case of pediatric TB should be considered alone [15, 16, 19]. Moreover children are the most vulnerable group for any health related problem. In addition there is very limited study done in Ethiopia, particularly in this study area on factors associated with the development of TB among children. Therefore this study was aimed to asses’ predictors of tuberculosis among children in public health facilities of Bale Zone, south east Ethiopia.

## Methods

### Study area and period

The study was conducted in public health institutions of Bale Zone, from January to June 2016.

Bale zone is one of the zones in Oromia Regional State and it comprises of 21 districts. Robe is an administrative town which is 423 km away from Addis Ababa, the capital city of Ethiopia. According to the 2013 population estimation, the total population of the zone was around 1,661,818 among this 791,024 (47.6%) were children below 15 years of age. In bale zone there are around 84 health centers and four hospitals that are providing DOTS both for adult and children [11].

### Study design

Institution based unmatched case control study was conducted among cases or children with TB and controls or children without TB in selected public health facilities of Bale Zone, south east Ethiopia.

### Population

#### Source and study population

All patients ≤ 15 years of age who came to public health facilities in Bale Zone were the source population and the study population wereall patients ≤ 15 years of age who came to the selected Public health Facilities.

#### Cases

All children ≤ 15 years of age who were on anti TB treatment. TB diagnosis was made based on the national comprehensive Tuberculosis, Leprosy, TB/HIV diagnosis and treatment manual [20]. The standard is applicable in every part of the country. Children who had TB but were attending OPD for health problems other than TB were excluded because one subject might have been selected twice since TB treatment is given in separate TB clinic.

#### Controls

All children ≤ 15 years of age who were on outpatient department and who were not diagnosed for TB.

### Exclusion criteria

Respondents who were critically ill during the data collection, those who come without parents were excluded because they were assumed unable to respond to the questions accordingly. Children who had the following symptoms during the immediate weeks before data collection, cough for more than 2 weeks duration, weight loss, night sweating and cervical & inguinal lymph node swelling were also excluded from cases and controls.. Those with the above symptom were excluded because we assume that diseases that have relatively the same symptoms with TB might be included in the case and might affect the study findings.

### Sample size and sampling procedure

#### Sample size determination

Sample size was calculated using Epi info version 3.5.1 software by employing proportion difference approach. Proportion difference approach is used because we assume that there is an exposure difference on risk factor among cases and controls. To detect whether the assumed difference exists between the two the following assumption were used. Since there were no study found in the study area and similar population: 1 to 2 cases to controls ratio was recruited to achieve 80% power and detect an odds ratio of 2.0 at 5% significance level if 20% or more of the general population was exposed to the risk factors at 95% confidence level. Then Total Estimated sample size was 411, (137 cases and 274 controls). Adding a 5% nonresponse rate, a total of 144 cases and 288 controls were the enrolment target.

#### Sampling procedures

As it is described in the study area section, there are 4 hospitals and 84 health centers that are providing DOTS service in Bale Zone. The 4 hospitals were purposively selected. In addition around 1/5th of the health centers (16 health centers) were randomly selected by first stratifying all the districts in to three agro ecological classification; Highland, Mid land and Low land. From Highland, Midland and Low land agro ecologic strata 5, 5 and 6 health centers were randomly selected respectively. Health centers were proportionally allocated to the number of health center in each agro ecologic strata.

To decrease the exposure disease relationship that can be due to the duration or prognosis of the disease, TB patients on intensive phase (patient on the first 8 weeks of treatment when the treatment is given under direct supervision of health care worker) were selected. For each case two consecutive controls that fulfill the inclusion criteria were sampled systematically from OPD of the same health facilities from which cases were drawn. Controls were selected by using systematic random sampling method. In order to determine the interval to select controls for each case, the 3 days average patient flow was fixed. The interval was calculated for every health facilities specifically.

### Data collection tools and procedures

Data were collected on the socio demographic, environmental, health related characteristics of the study participants. Semi structured questionnaires were used to collect the data. Most of the questions were adapted and modified from previous study and reviewing of different literatures [12, 21, 13–15, 22]. The questionnaires were first developed in English and were translated in to local language (Amharic and Afaan Oromo) and were translated back to English. Review was made by Amharic, English, Afaan Oromo language experts and health professionals for consistency of translation of the language. Data was collected using face to face interview methods with the parents of children. Both cases and controls were interviewed by a person working on TB clinic of respective health facility after confirming their medical diagnosis from clients’ medical card. The data collectors conducted the interview after providing respondents a brief orientation on the purpose of the study and its significance.

### Data quality control

Before data collection, pretest was be done in 5% of the sample size population in Robe health center which were not included in the actual sampling before the actual data collection period and necessary adjustments were made on the tool.

Three days training was given to data collectors and supervisors about the objectives of the study, data collection instruments, data collection procedures, and the ethical consideration during data collection. The investigators supervised and reviewed every questionnaire for completeness and logical consistency and corrections were made at the data collection site. Data coding, entry and cleaning was performed by the investigators.

### Data processing, analysis and presentation

The data was checked for completeness and consistencies, and cleaned, coded and entered using Epi Info version 3.5.4 and was exported to statistical package for social sciences (SPSS) software version 20 for analysis. Descriptive statistics was performed to describe the study population in relation to relevant variable and findings were presented as proportions.

Multiple logistic regression model was fitted to find factors associated with pediatrics TB. The candidate variables considered were age of the child, religion, ethnicity, educational status of the mother, educational status of the father, family size, availability of separate kitchen and window, family practice of feeding children raw milk, BCG vaccination status, HIV status of the child, family history of TB, occupation of the father, occupation of the mother and family size. BCG vaccination status of the children was assessed based on the presence of scar the right upper arm. In addition, the national average individual size per household was used to classify family size.

Firstly all variables were screened by carrying-out univariable analyses using a liberal *p-value* of 0.20. All variables significant at the 20% level were included in the multivariable analysis control confounders and identify independent effect of different factors for occurrence of TB. Afterwards, stepwise logistic regression model was used to remove variables not significant at 5% significance level. The goodness of fit test was investigated using Hosmer and Lemeshow good-ness of fit test. All analyses were done using SPSS version 20.

## Result

A total of 432 children from 20 health facilities were selected to participate in the study, of which 426 participated in the study which gives 98.6% response rate.

### Socio-demographic characteristics

Among the total 426 participants 142 were cases and 244 were controls. There were equal number of male and female 71 (50%) among cases and controls. Among the controls, 136 (47.9%) and 148 (52.1%) were males and females, respectively. The mean (standard deviation) of age among cases was 8.4 (±4.3) and it was 7.3 (±4.1) among controls.

With regard to occupational status of the mother among cases, 70 (49.3%) were housewives and 8 (5.6%) were government employees, while among controls 107 (37.7%) were housewives and 47 (16.5) were government employed (Table 1).

**Table 1:** Socio demographic characteristics of pediatric patient with TB and Without TB in public health facilities of Bale zone, south east Ethiopia, 2016

Variable	Level	Disease status	
		Case	Control
		Frequency (%)	Frequency (%)
Age	≤5	39 (27.5)	106 (37.3)
	5–10	50 (35.2)	105 (36.9)
	10–15	53 (37.3)	73 (25.7)
Sex	Male	71 (50)	136 (47.9)
	Female	71 (50)	148 (52.1)
Religion	Muslim	102 (71.8)	142 (50)
	Orthodox	32 (22.5)	109 (38.4)
	Protestant	2 (1.4)	30 (10.6)
	Catholic	2 (1.4%)	2 (0.7)
	Wakefeta	4 (2.8%)	1 (0.4)
Ethnicity	Amhara	9 (6.3%)	62 (21.8)
	Oromo	121 (85.2%)	178 (62.7)
	Tigre	2 (1.4%)	13 (4.6)
	Gurage	2 (1.4%)	14 (4.9)
	Others	8 (5.6%)	17 (6.0)
Child’s educational status	Can’t read and write	13 (9.2%)	5 (1.8)
	Not enrolled	54 (38.0%)	135 (47.5)
	Primary school	68 (47.9%)	140 (49.3)
	Secondary school	7 (4.9%)	4 (1.4)
Mother’s educational status	Can’t read and write	69 (48.6%)	59 (20.8)
	Can read and write	29 (20.4%)	46 (16.2)
	Primary school	20 (14.1%)	71 (25.0)
	Secondary education	19 (13.4)	74 (26.1)
	Above secondary education	5 (3.5)	34 (12.0)
Father’s educational status	Can’t read and write	39 (27.5)	35 (12.3)
	Can read and write	45 (31.7)	46 (16.2)
	Primary school	26 (18.3)	48 (16.9)
	Secondary education	20 (14.1)	86 (30.3)
	Above secondary education	12 (8.5)	69 (24.3)
Mother’s occupation	Farmer	34 (23.9)	27 (9.5)
	Merchant	25 (17.6)	83 (29.5)
	Gov’t employee	8 (5.6)	47 (16.5)
	Non gov’t employee	2 (1.4)	15 (5.3)
	Day labor	3 (2.1)	5 (1.8)
	Housewife	70 (49.3)	107 (37.7)
Father’s occupation	Farmer	82 (57.7)	73 (25.7)
	Merchant	32 (22.5)	77 (27.1)
	Gov’t employee	13 (9.2)	84 (29.6)
	Non gov’t employee	6 (4.2)	28 (9.9)
	Day labor	3 (2.1)	13 (4.6)
	Pastoral	2 (1.4)	3 (1.1)
	Others	4 (2.8)	6 (2.1)
Family size	≤5	65 (45.8)	185 (65.1)
	> 5	77 (54.2)	99 (34.9)

### Environmental and house hold factors

From a total of 426 participants 123 (86.6%) of cases and 226 (79.4%) controls have privately owned house. Around 41 (28.9%) and 39 (13.7%) of cases and controls, respectively, has had more than six family members. According to this study, the availability and functionality of windows among cases and controls were 120 (84.5%) among which 109 (90.8%) were functional and 266 (93.7) among which 257 (96.6%) were functional respectively. With regard to availability of separate kitchen for cooking, 108 (76.1%) of cases and 253 (89.1%) of controls have separate kitchen for cooking.

Among a total of 142 cases, majority 105 (73.9%) of them have a habit of feeding raw milk to their child whereas only 86 (30.3%) of controls have a habit of feeding raw milk to their children.

### Clinical characteristics of TB and co-morbid illness

Of the total study participants, 12 (8.5%) of cases had previous history of TB whereas only 6 (2.1%) of controls had previous history of TB.

When asked about recent family history of TB, while 45 (31.7%) of the cases responded positively only 10 (3.5%) said they have had family history of TB. More than a quarter 39 (27.5%) of the cases and 7 (2.5%) of the controls were not vaccinated for BCG.

Co-morbid illnesses that are related with pediatric TB were also assessed. Our study revealed that none of the cases were diabetic whereas 5 (1.8%) of the controls had history of raised blood glucose level (BGL). Regarding HIV status of the study participants, 16 (11.3%) of the cases and 4 (1.4%) of the controls were infected with HIV (Table 2).

**Table 2:** Logistic regression analysis of factors associated with pediatric TB in public health facilities of Bale Zone, south east Ethiopia, 2016

Variable	Level	Disease status		COR (95% CI)	AOR (95% CI)
		Control	Case		
		Frequency (%)	Frequency (%)		
Sex	Male	136 (47.9)	71 (50.0)	1	
	Female	148 (52.1)	71 (50.0)	1.08(0.72–1.63)	–
Age	≤5	106 (37.3)	39 (27.5)	1	1
	6–10	105 (37.5)	50 (35.2)	1.29(0.78–3.18)	1.26(0.67–2.43)
	11–15	73 (25.7)	53 (37.3)	1.97(1.18–3.18)	1.94(1.02–3.77)
Mother’s educational status	Can’t read and write	59 (20.8)	69 (48.6)	7.95(2.92–21.64)	2.35(0.68–5.05)
	Can read and write	46 (16.2)	29 (20.4)	4.28(1.50–12.22)	1.54(0.43–5.49)
	Primary	71 (25.0)	20 (14.1)	1.91(0.66–5.53)	1.68(0.49–5.72)
	Secondary	74 (26.1)	19 (13.4)	1.76(0.60–5.06)	1.14(0.62–2.17)
	Tertiary	34 (12.0)	5 (3.5)	1	
Family size	≤5	185 (65.1)	65 (45.8)	1	
	> 5	99 (34.9)	77 (54.2)	2.21(0.46–3.33)	–
No of people living in the house	≤3	44 (15.5)	13 (9.2)	1	
	4–6	201 (70.8)	88 (62.0)	1.48(0.76–2.88)	1.55(0.63–3.81)
	> 6	39 (13.7)	41 (28.9)	3.56(1.66–7.56)	1.75(0.55–5.57)
Number of rooms in the house	1–3	254 (89.4)	136 (95.8)	1	
	4–6	30 (10.6)	6 (4.2)	0.37(0.15–1.92)	–
Availability of windows	Yes	266 (93.7)	120 (84.5)	1	
	No	18 (6.3)	22 (15.5)	2.7(1.40–5.32)	0.85(0.33–2.16)
Availability of separate kitchen	Yes	253 (89.1)	108 (76.1)	1	
	No	31 (10.9)	34 (23.9)	2.56(1.50–4.39)	0.53(0.20–1.22)
Availability of latrine	Yes	252 (88.7)	113 (79.6)	1	
	No	32 (11.3)	29 (20.4)	2.02(0.99–3.50)	–
Waste disposal	In the compound	97 (34.2)	78 (54.9)	2.35(1.56–3.55)	1.03(0.59–1.81)
	Outside compound	187 (65.8)	64 (45.1)	1	
Availability of tap water	Yes	186 (65.5)	50 (35.2)	1	
	No	98 (34.5)	92 (64.8)	3.49(2.29–5.33)	1.27(0.66–2.44)
Do animal live in your house	Yes	34 (12.0)	54 (38.0)	4.51(2.76–7.39)	1.75(0.86–3.56)
	No	250 (88.0)	88 (62.0)	1	
Do you feed your child row meat	Yes	204 (71.8)	115 (81.0)	1.67(1.01–2.73)	–
	No	80 (28.2)	27 (19.0)	1	
Do you feed your child raw milk	Yes	86 (30.3)	105 (73.9)	6.53(4.16–10.27)	4.23(2.26–7.88)
	No	196 (69.7)	37 (26.1)	1	
Previous history of TB	Yes	6 (2.1)	12 (8.5)	4.27(0.96–11.65)	–
	No	278 (97.9)	130 (91.5)	1	
BCG vaccination status	Yes	277 (97.5)	103 (72.5)	1	
	No	7 (2.5)	39 (27.5)	13.52(6.13–29.88)	5.46(1.82–16.32)
Family history of TB	Yes	10 (3.5)	45 (31.7)	12.71(6.17–26.20)	9.84(4.22–22.92)
	No	274 (96.5)	97 (68.3)	1	
Vaccination at 9 month	Yes	254 (89.4)	87 (61.3)	1	
	No	30 (10.6)	55 (38.7)	5.35(3.22–8.89)	0.98(0.44–2.19)
Resent history of diarrheal disease	Yes	51 (18.0)	16 (11.3)	0.58(0.32–1.06)	–
	No	233 (82.0)	126 (88.7)	1	
Resent history of hospitalization	Yes	37 (13.0)	31 (21.8)	1.86(1.01–3.16)	–
	No	247 (87.0)	111 (78.2)	1	
HIV status of the child	Positive	4 (1.4)	16 (11.3)	8.89(2.91–27.12)	13.61(3.45–53.69)
	Negative/unknown	280 (98.6)	126 (88.7)	1	
Does the child has any chronic disease	Yes	14 (4.9)	12 (8.5)	1.78(0.8–3.96)	–
	No	270 (95.1)	130 (91.5)	1	

### Factors associated with pediatrics TB

For the multivariable logistic regression, the variables that were found to be significant in the univariable analysis were considered. Sex of the child, family size, number of rooms in the house, availability of latrine, consumption of raw meat, previous history of TB, recent history of diarrhea and hospitalization, and presence chronic disease were found not to be statistically significant. Age of the child, mother’s educational status, number of people residing in the house, availability of windows and separate kitchen, presence of waste disposal, presence of animals living in the house, consumption of raw milk, BCG vaccination status and family history of TB were found to be statistically associated with pediatrics TB. The results of the logistic regression are presented in Table 2. Higher odds of pediatrics TB was associated with age group 11–15 compared children less than or equal to 5 years (odds ratio (OR) 1.94 95% C.I. 1.02–3.77).

Another important variable that shows strong association was family history of TB. The odds of TB among those who had recent family TB history was 10 times higher among those who didn’t have family history of TB with AOR 9.84 95% C.I. (4.22–22.92).

Higher odds of pediatrics TB was also associated with child being infected with HIV compared to those who were uninfected (AOR = 13.6, 95% C.I: 3.45–53.69); those children who were fed raw milk compared to those who were not (AOR = 5.46, C.I: 1.82–16.32). Higher odds of TB were also associated with being vaccinated for BCG vaccine compared with absence of BCG vaccination (AOR = 5.46, 95% C.I: 1.82–16.32).

## Discussion

This case control study assesses predictors for the occurrence of TB among children in public health facilities. Age of the child, contact history with TB patient, absence of BCG vaccination, presence HIV infection and consumption of raw cow milk were independently associated with increased risk of pediatrics TB.

Despite previous perceptions that TB in pediatrics age do not pose greater risk, since children tend to acquire mild disease, contribute less to transmission to other people and do not impact epidemic control, pediatrics TB has been considered as an important public health problem which is easily preventable cause of disease and death.

Our study identified several risk factors that contribute to the development of pediatrics TB. Some of these factors have also been previously described [23, 24, 25, 26], and confirmed by our study. Studies indicated that various factors contribute to the development of pediatrics TB. The findings of our study indicated that the age of the child was one of the predictor for pediatric TB, in which the risk was higher among 11–15 age group children when compared with children of age group ≤5. This finding was supported by Federal Ministry of Health 16th National Annual review Report on Tuberculosis*,* proportion of TB patients among older children aged 5–14 were higher than those aged 0–4 years [27]. This could be explained by the fact that as the age of the child increase, the probability of the child to spend time outside his/her home is high which in-turn increases their chance of contact with infectious cases.

Effective investigation of TB contacts is of great importance in that they contribute greatly to early detection of active TB, thus decreasing its severity and transmission to others. Our study unsurprisingly revealed that TB disease has a ten-fold increase among study participants with contact history with TB patients. This finding was confirmed by several previous studies conducted elsewhere [23, 28, 29, 30], where they indicated contact history as an important risk factor for the transmission of childhood TB. In most low- and middle-income settings, even-though investigation of TB contact is included in the national TB control program, it is rarely and consistently conducted due to resource constraints. However, contact history investigation merits serious consideration as a means to improve early case detection and prevent transmission of mycobacterium tuberculosis infection.

In our study, family history of feeding children raw cow milk was strongly associated with pediatric TB where children who has history of consuming raw milk had 4 folds increase in the odds of developing TB than those who don’t, where previous study in Russia [24] indicated similar finding. The association between consumption of raw milk and TB in this study can be explained the fact that TB can also be caused by *mycobacterium bovis* which is found in unpasteurized milk. The consumption of unpasteurized milk in the study area is high. If the association proves to be due to *mycobacterium bovis,* which the current study has not identified, ensuring milk safety would be a public health priority.

In high HIV prevalence settings, tuberculosis (TB) and HIV/AIDS cannot be considered in isolation from each other. Co-infection with HIV, especially in sub-Saharan countries, has significantly increased the number of individuals with TB. TB is the leading cause of death in HIV positive people, and HIV infection is a significant risk factor for TB [31]. Our study found that HIV infected children are more likely to be infected with TB.

Similarly, Investigators from a case control study conducted in Zambia [32], a study in Northwest Ethiopia [17] and another study from South Africa [33] reported that TB was associated with the increased HIV infection. The current study also revealed that children who were vaccinated for BCG have a reduced risk of TB. This was evidenced by meta-analysis of available studies where BCG vaccine can reduce risk of TB infection by as much as 50% [26].

### Strengths

We used case control study design which is appropriate to address the research question. The cases and controls were also taken from the same institutions which made it easy to identify controls.

### Limitations

One of the limitations of this study might be recall bias as study participants may not remember condition of the past. Additional limitation was environmental and house hold factors were assessed without observation and this may have underestimated or overestimated the effect in the findings. Although malnutrition is important factor that highly contribute for the occurrence of TB in children, we were unable to analyze it due to incompleteness of most of the data.

## Conclusion

The finding of this study has given insight on different predictors of pediatric TB. Children that have contact history with adult individual with TB are at risk of acquiring the infection. BCG vaccination status, HIV status, age of the child and family practice of feeding children raw milk are the independent predicators of pediatric TB. Therefore Bale Zonal health office is highly recommended to work with different stakeholders to increase public awareness on risk factors of pediatric TB and its transmission. Contact tracing and contact screening should be correctly implemented to prevent transmission of the infection and early diagnosis. Further research should be done to investigate if bovine TB is dominant in the area to take appropriate measure.

## Abbreviations

AIDS: Acquired immune deficiency syndrome
BZHB: Bale zone health bureau
CI: Confidence interval
DOTS: Directly observed treatment, short course
EPI: Expanded program of immunization
EPTB: Extra-pulmonary tuberculosis
FMOH: Federal Ministry of Health
HIV: Human immunodeficiency virus
MDR-TB: Multidrug resistant tuberculosis
MWU: MaddaWalabu University
OR: Odds Ratio
PTB: Pulmonary tuberculosis
TB: Tuberculosis
TB/HIV: Tuberculosis and HIV co-infection
TBIC: Tuberculosis infection control
WHO: World Health Organization
XDR-TB: Extensively drug resistant tuberculosis

## References

[1] World Health Organization Fact Sheet No.104: Tuberculosis. Geneva: WHO; 2010. Available from: http://www.who.int/mediacentre/factsheets/fs104/en/print.html.

[2] Fauci B, Kasperetal H. Principle of Internal Medicine. United States of America: McGraw-Hill Medical Publishing Devision; 2008. 17^th^edition, chapter 38.

[3] WHO (2015). Global tuberculosis report.

[4] WHO (2014). Global tuberculosis report.

[5] FMOH. Ethiopian Population Based National TB Prevalence Survey Research Protocol. Addis Ababa: FMOH; 2010.

[6] WHO (2014). Global tuberculosis report. Library cataloguing-in-publication data.

[7] Dodd PJ (2017). The global burden of tuberculosis mortality in children: a mathematical modeling study. Lancet Glob Health.

[8] WHO (2016). Global tuberculosis report 2016.

[9] Tsai K, Chang H (2013). Childhood tuberculosis: epidemiology, diagnosis, treatment and vaccination. Pediatr Neonatol.

[10] Jamie C, Jessica G (2009). Assessing Tuberculosis Management and Prevention in Rural and Semi-urban Ethiopia.

[11] Bale Zone Health Bureau (2015). Annual report.

[12] Anamarija J (2013). Risk factors for pulmonary tuberculosis in Croatia: a matched case control study. BMC Public Health.

[13] Kirenga BJ, et al. Tuberculosis risk factors among tuberculosis patients in Kampala, Uganda: implications for tuberculosis control. BMC Public Health. 2015;15:13. 10.1186/s12889-015-1376-3. 10.1186/s12889-015-1376-3 PMC4311451 25604986

[14] Mohamed R, Mohamed A (2012). Risk factors of childhood tuberculosis in rural Bangladesh. WHO South-East Asia J Public Health.

[15] Kefyalew T. Risk Factors for Active Tuberculosis in Children in Contact with Smear Positive Cases in Southern Ethiopia: Maters Thesis University of Liverpool; 2010. p. 57–9. http://www.academia.edu/2492196/.

[16] Cheru T, Takele T (2015). Environmental and host-related determinants of tuberculosis in Metema district, north-West Ethiopia. Drug Healthcare Patient Saf.

[17] Moges B (2012). Prevalence of smear positive pulmonary tuberculosis among prisoners in North Gondar zone prison, Northwest Ethiopia. BMC Infect Dis.

[18] Muñoz-Sellart M (2009). Treatment outcome in children with tuberculosis in southern Ethiopia. Scand J Infect Dis.

[19] Ramos A (2010). Childhood and adult tuberculosis in a rural hospital in Southeast Ethiopia: a ten-year retrospective study. BMC Public Health.

[20] FMOH (2016). Ethiopia National comprehensive Tuberculosis, Leprosy, TB/HIV diagnosis and treatment manual.

[21] Marais B (2014). The natural history of childhood intra thoracic tuberculosis, a critical review of the pre-chemotherapy literature. Cold Spring HarbPerspect Med.

[22] FMOH (2011). Ethiopian National Population Based Tuberculosis Prevalence Survey.

[23] Karim M (2012). What cannot be measured cannot be done; risk factors for childhoodtuberculosis: a case control study. Bangladesh Med Res Counc Bull.

[24] Coker R (2006). Risk factors for pulmonary tuberculosis in Russia: case-control study. BMJ.

[25] Singh M (2005). Prevalence and risk factors for transmission of infection among children in household contact with adults having pulmonary tuberculosis. Arch Dis Child.

[26] Colditz GA (1994). Efficacy of BCG vaccine in the prevention of tuberculosis meta-analysis of the published literature. JAMA.

[27] FMOH (2014). Evaluating the National TB Control Program: Challenges and ways forward Disease Prevention and Control. 16th National Annual Review Meeting Group Discussion.

[28] Simon S, Ben JM (2007). Culture-confirmed childhood tuberculosis in cape town, South Africa: a review of 596 cases. BMC Infect Dis.

[29] Jurcev-Savicevic A (2013). Risk factors for pulmonary tuberculosis in Croatia: a matched case– control study. BMC Public Health.

[30] Morrison J, Pai M, Hopewell PC (2008). Tuberculosis and latent tuberculosis infection in close contacts of people with pulmonary tuberculosis in low-income and middle-income countries: a systematic review and meta-analysis. Lancet Infect Dis.

[31] Ashley K (2012). TB, HIV, and TB/HIV co-infection: community knowledge and stigma in western Uganda by maters thesis.

[32] Boccia D, Hargreaves J, De Stavola BL, Fielding K, Schaap A, Godfrey-Faussett P, et al. (2011) The association between household socioeconomic position and prevalent tuberculosis in Zambia: a case-control study. PLoS One 6(6): e20824. https://doi.org/10.1371/journal.pone.0020824 10.1371/journal.pone.0020824 PMC3117783 21698146

[33] Hesseling AC (2009). High incidence of tuberculosis among HIV-infected infants: evidence from a south African population-based study highlights the need for improved tuberculosis control strategies. Clin Infect Dis.

